# A role for SNX5 in the regulation of macropinocytosis

**DOI:** 10.1186/1471-2121-9-58

**Published:** 2008-10-14

**Authors:** Jet Phey Lim, Jack TH Wang, Markus C Kerr, Rohan D Teasdale, Paul A Gleeson

**Affiliations:** 1 The Department of Biochemistry and Molecular Biology and Bio21 Molecular Science and Biotechnology Institute, The University of Melbourne, Melbourne, Victoria 3010, Australia; 2 Institute for Molecular Bioscience, The University of Queensland, Brisbane, Queensland 4072, Australia

## Abstract

**Background:**

The mechanisms and components that regulate macropinocytosis are poorly understood. Here we have investigated the role of sorting nexin 5 (SNX5) in the regulation of macropinocytic activity.

**Results:**

SNX5 is abundantly expressed in macrophages, cells very active in macropinocytosis, and is recruited onto newly-formed macropinosomes. LPS treatment of bone marrow-derived macrophages resulted in a 2.5 fold decrease in macropinosome formation that correlates with a reduction in the levels of SNX5. To investigate the relationship between SNX5 levels and macropinocytic activity we examined the formation of macropinosomes in HEK-FlpIn cells stably expressing GFP-SNX5. Constitutive macropinocytosis was increased ~2 fold in HEK-GFP-SNX5 cells compared with parental HEK-FlpIn cells. Furthermore, EGF stimulation resulted in a significant increase in macropinocytosis and there was also a 2.0 fold increase in the generation of macropinosomes in HEK-GFP-SNX5 cells compared with parental HEK-FlpIn cells. SNX5, which interacts specifically with PtdIns(3)*P *and PtdIns(3,4)*P*_2 _through its PX domain, was recruited to regions on the plasma membrane containing EGF receptor or positive for PtdIns(3,4)*P*_2 _as detected with the PH domain of TAPP1. Treatment with AG1478, an EGF receptor specific tyrosine kinase inhibitor, prevented the recruitment of SNX5 to the cytosolic face of the plasma membrane and inhibited the formation of macropinosomes in response to EGF treatment.

**Conclusion:**

Based on these data, we propose that SNX5 requires the generation of phosphoinositides for recruitment to the plasma membrane and, moreover, influences the level of macropinocytic activity.

## Background

Macropinocytosis is an endocytic process that enables cells to internalize large amounts of solutes from the external environment. Macropinosomes are generated from the base of actin-mediated membrane ruffling when the lamellipodia folds back onto itself thereby forming very large endocytic structures. Macropinosomes are heterogeneous in size and generally considered to be > 0.2 μm in diameter [1,2], a size considerably larger than clathrin-coated vesicles. The formation of macropinosomes is largely a signal dependent process that is transiently induced by growth factors such as macrophage colony-stimulating factor (M-CSF) and epidermal growth factor (EGF) or tumour promoting factors such as phorbol myristate acetate (PMA) [3-6]. Given the large size of macropinosomes, this unique organelle provides an efficient route for non-selective entry of solute macromolecules as well as considerable amounts of plasma membrane into the cell [2].

Macropinocytosis is important in a range of physiological processes. For example, macropinocytosis has a role in the down-regulation of signalling from the plasma membrane [7] and, because of its dependence upon membrane ruffling, in cell motility [2]. Consequently macropinocytosis is very relevant to tumour progression and metastasis. In addition, this endocytic pathway is the primary mechanism by which macrophages and dendritic cells sample their immediate environment for circulating antigens [8]. Indeed, the major antigen presenting cells, namely macrophages and dendritic cells, are highly active in macropinocytosis [8]. For example, macrophages undergo extensive constitutive macropinocytosis, internalizing up to 200% of their surface area every hour [9], patrolling and sampling the environment for their role as antigen presenting cells of the immune system. Also immature dendritic cells are able to macropinocytose large quantities of exogenous solute as part of their sentinel function [10]. On the other hand, maturation of dendritic cells is associated with down-regulation of macropinocytosis to maximise the presentation of captured antigen [11]. In addition to antigen uptake, macropinocytosis is also considered important in the chemotactic response of neutrophils and macrophages [12]. This endocytic pathway is also utilised by various pathogens such as *Salmonella *and *Shigella *to gain entry into host cells [13].

Despite the physiological relevance of macropinocytosis, the molecular basis for the regulated formation and maturation of macropinosomes is very poorly understood. Macropinosome formation in a range of cell types has been shown to be phosphoinositide-3 kinase dependent [14] and unlike the relatively well-characterised phagosome, its regulation is receptor-mediated. As endocytic compartments mature, the bulk of their protein constituents are maintained [15]. Peripheral membrane proteins are differentially recruited in a temporally dependent manner in response to a shift in the organelle's phosphoinositide composition. The phosphoinositides (PtdIns) have become the focus of intense interest as they are linked to a range of cell signalling events and are key regulators of intracellular membrane trafficking. Whilst PtdIns(3,4)*P*_2 _[16], PtdIns(4,5)*P*_2 _[17] and PtdIns(3,4,5)*P*_3 _[18] are traditionally considered to be associated with signalling at the plasma membrane in response to extracellular stimuli, the monophosphorylated phosphoinositide PtdIns(3)*P*, is implicated in the membrane trafficking of the endosomal system.

Sorting nexins are a large family of proteins characterised by the presence of a phox (PX) domain at the amino terminus. The modestly conserved PX domain is a sequence of 70 to 120 residues that has been shown to bind to various phosphoinositides hence the PX domain confers phosphoinositide specificity to the protein [19]. Sorting nexins have roles in endocytic trafficking events [19-21]. One such sorting nexin is SNX5, a 404 residue protein that contains a central PX domain and large C-terminal domain predicted to include a BAR domain [22,23], a domain believed to be involved in membrane curvature and tubulation. SNX5 was first discovered based on its homology to SNX1 and subsequently shown to associate with early endosomes. We have previously reported that the PX domain of SNX5 binds specifically to PtdIns(3)*P *and PtdIns(3,4)*P*_2 _[24] and SNX5 is associated with newly-formed macropinosomes following EGF stimulation [25]. After EGF stimulation of HEK293 cells, large EEA1- and SNX5-positive structures were detected in close proximity to membrane ruffles which labelled with fluid phase markers [25], characteristics that define macropinosomes. We have also exploited the association of SNX5 with macropinosomes to track their maturation in live cells [25]. Our previous work showed that the association of SNX5 with macropinosomes is highly dynamic and SNX5 is localised not only to the macropinosome body but also tubules that extend and depart from the newly-formed macropinosomes [25]. Here, we present data showing that SNX5 modulates macropinocytic activity as well as evidence that the generation of phosphoinositides is involved in the recruitment of SNX5 to the plasma membrane prior to macropinosome formation.

## Methods

### Antibodies, conjugates and DNA constructs

Rabbit anti-mouse SNX5 was raised against a synthetic peptide corresponding to the N-terminal 35 residues of mouse SNX5, with an additional Cys on the C-terminus, and affinity purified on a column of peptide conjugated to SulfoLink Coupling Gel (Pierce, USA). Monoclonal mouse antibodies to human EEA1 were from BD Transduction Laboratories (California, USA). Mouse monoclonal anti-human EGF receptor (EGF-R) antibody (clone 528) has been described [26]. Monoclonal mouse antibodies to α-tubulin, goat anti-rabbit IgG Alexa Fluor^® ^488, goat anti-mouse IgG Alexa Fluor^® ^568, goat anti-mouse IgG Alexa Fluor^® ^647, biotinylated EGF complexed to Alexa Fluor^® ^555 streptavidin and Texas-Red-X Phalloidin were purchased from Molecular Probes (Oregon, USA). Horse-radish peroxidase conjugated rabbit anti-mouse and swine anti-rabbit immunoglobulins were from Dako (Glastrup, Denmark). Full length human EGF-R was cloned into the XbaI sites of pCI-neo. The PH domain of TAPP1 (aa 95–400) was from Dr. S. Dowler (MRC, University of Dundee, Dundee, UK).

### Cell culture and transfection

HEK-FlpIn (Invitrogen, USA) and HEK-GFP-SNX5 stable cells were generated, cultured and transfected as previously described [24]. Primary bone marrow-derived macrophages (BMMs) were generated by culture of bone marrow cells for 7 days in M-CSF as described [27]. BMMs were activated with 10 ng/ml LPS (Sigma-Aldrich, Australia) up to 24 h before fixation in 4% paraformaldehyde (PFA) or preparation for immunoblotting.

### Macropinosome analyses

Cell monolayers were cultured for various time periods in the presence of 100 μg/ml dextran (10,000 MW) conjugated to tetramethylrhodamine (TR-dextran) (Molecular Probes, USA). The cells were then washed with media at 4°C and fixed in 4% PFA. Images were captured using an LSM 510 Meta confocal microscope and quantified for macropinosomes using an automated image analysis pipeline in ImageJ 1.37v (NIH). The 'Substract Background' functionality was executed, and images thresholded to a binary image. Dextran-positive structures at least 0.5 μm in diameter were quantified through the 'Analyze Particles' feature.

For analysis of macropinosomes using endosomal markers, cell monolayers were washed twice using PBS, serum-starved for 16–24 h at 37°C and stimulated with EGF (100 ng/ml) (Invitrogen, USA) at 37°C for 10 min. Cells were then washed twice in PBS, fixed in 4% PFA and stained using mouse anti-human EEA1 followed by goat anti-mouse Alexa Fluor 568. Macropinosomes were identified based on their size (>1 μm) [2], circular structure and the staining pattern for EEA1 which resembled a halo. The mean number of macropinosomes from a triplicate of 100 cells was determined. Each experiment was repeated at least twice without knowledge of the identity of samples. Counts were performed using the Zeiss Axioplan 2 microscope.

### EGF Internalization assay

HEK-GFP-SNX5 cells, transiently transfected with pCI-neo-EGF-R for 24 h, were serum-starved for 16–24 h at 37°C. Cells were washed twice using ice-cold PBS and incubated with 100 ng/ml Alexa Fluor 555 conjugated-EGF (Molecular Probes, USA) in serum-free media for 10 min on ice. Excess EGF was removed by washing the cells three times in ice-cold PBS. Cells were resuspended in ice-cold serum-free media (in the absence and presence of drugs) and the cells were then incubated at 37°C for the indicated duration to allow EGF internalization. Cells were then fixed and processed for immunofluorescence analysis.

### Drug treatment

Cell monolayers were washed twice with PBS and serum-starved for 16–24 h at 37°C. Cells were treated with 100 nM AG1478 (Calbiochem, USA) (from a 10 mM stock in DMSO) for 15 min at 37°C. EGF (100 ng/ml) (Invitrogen, USA) was then added to the cells in the presence or absence of AG1478 at 37°C for the indicated durations. Cells were then washed twice with PBS and processed for immunofluorescence.

### Indirect immunofluorescence

Cell monolayers grown on poly-L-lysine coated coverslips were washed twice in PBS and fixed in 4% PFA for 15 min. Cells were processed for immunofluorescence as previously described [28] and examined using either a Zeiss Axioplan 2 microscope or a Leica TCS SP2 laser unit and Leica Confocal Software version 2.61 or a Zeiss LSM 510 Meta confocal microscope. For multi-colour labelling, images were collected independently.

### Endosome-lysosome fusion assay

Late endosomes and lysosomes of cells were labelled with fluorescently-conjugated dextran as described previously [29]. Briefly, cells grown on poly-L-lysine coated coverslips were incubated with 100–200 μg/ml dextran conjugated to Alexa 647 (MW 10,000 Da) (Molecular Probes, USA) for 4 h at 37°C. The cells were then thoroughly washed and further incubated in normal growth media for 16 h to allow the dextran to accumulate in the late organelles of the endosomal system. The cells were then pulsed with 100–200 μg/ml dextran conjugated to TR (MW 10,000 Da) (Molecular Probes, USA) for 5 min at 37°C to label macropinosomes, washed and chased in normal media for 4, 10, 15 or 20 min before being fixed in 4% PFA. The cells were imaged on an LSM 510 Meta confocal microscope. The proportion of TR-labelled macropinosomes that had acquired content from the late endosomes/lysosomes (ie. Alexa 647-positive) were scored for >50 cells per time point.

### Immunoblotting

Proteins were resolved on 10% SDS-polyacrylamide gels and transferred onto Immobilon-P PVDF membranes (Millipore, USA) according to the manufacturer's instructions and immunoblotting performed as described [30]. SNX5 was visualized with rabbit anti-SNX5 followed by horseradish peroxidase-conjugated anti-rabbit secondary antibody. Bound antibodies were detected using chemiluminescence and images were captured using Gel-Pro™ Analyser version 4.5 software. Bound antibodies were stripped from the membrane using PVDF membrane stripping buffer (25 mM glycine, 1% (w/v) SDS, PBS, pH 2) as necessary. Membrane was then washed and blocked, as described above, prior to subsequent incubation with different primary antibodies.

### Statistical Analysis

Data were analysed by an unpaired Student's *t*-test, two-tailed.

## Results

### SNX5 is located on macropinosomes in both cultured and primary cells

**Figure 1:**
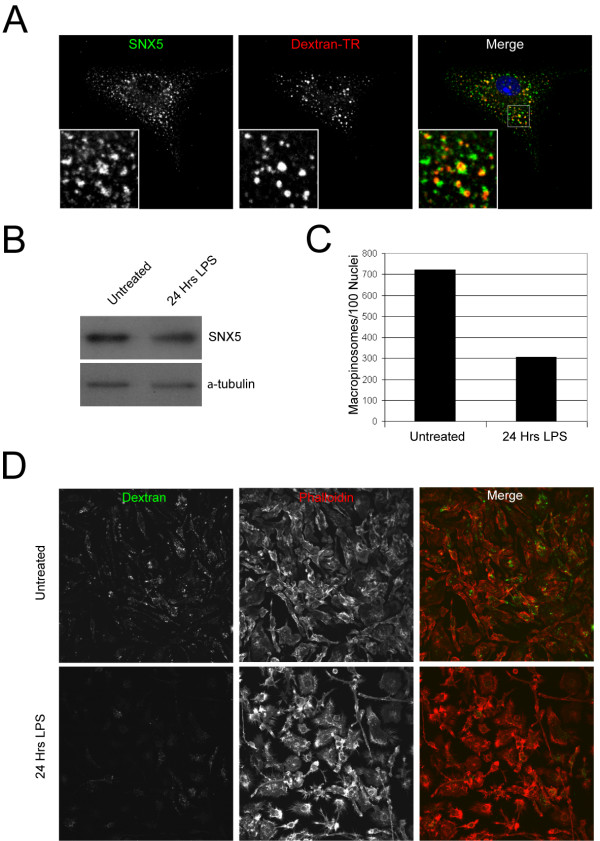
**LPS stimulation of primary macrophages results in a decrease in macropinocytic activity and SNX5 levels**. (A-D) Bone marrow-derived macrophages were grown for 7 days in M-CSF and then analysed as follows. (A) Cells were cultured in the presence of 100 μg/ml TR-conjugated dextran (10,000 MW) for 3 min. The cells were washed at 4°C and fixed in 4% PFA and stained with affinity-purified SNX5 antibodies followed by Alexa488-conjugated goat anti-rabbit IgG. Bar = 10 μm. (B) Macrophages were seeded at 1 × 10^7 ^cells/ml and incubated with 10 ng/ml LPS for up to 24 h, as indicated. Samples (15 μg protein) were separated by SDS-PAGE and probed by immunoblotting using the anti-SNX5 antibody. (C-D) Macrophages were either left untreated or treated with 10 ng/ml LPS for 24 h and then pulsed with 1 mg/ml Alexa 488-conjugated dextran for 3 minutes at 37°C and fixed in 4% PFA. The samples were then stained with Texas-red-X conjugated phalloidin. Images were captured using identical settings for -/+ LPS conditions and counted as described in Methods. At least 180 cells were analysed for each condition.

We have previously shown that GFP-SNX5 is associated with macropinosomes in HEK-FlpIn cells [25]. The question now arises as to whether SNX5 is also a marker of macropinosomes in primary cells and, if so, what function SNX5 plays in the process of macropinocytosis. Macrophages are known to have very high rates of macropinocytosis [31]. Significantly, microarray data has indicated that macrophages and immature dendritic cells express very high levels of SNX5 transcripts [32] coinciding with their high macropinocytic activities. Therefore, we examined if SNX5 is a marker of macropinosomes in macrophages. Analysis of the intracellular localization of endogenous SNX5 in bone marrow-derived mouse macrophages (BMMs) differentiated for 7 days in M-CSF revealed that endogenous SNX5 is associated with endosomes and large intracellular structures reminiscent of macropinosomes (Fig 1A). These large intracellular structures (0.5–5 μm) were readily labelled after a 3 min pulse of TR-conjugated dextran confirming them as newly-formed macropinosomes. Furthermore, BMMs were analysed by immunoblotting using an affinity purified rabbit polyclonal antibody to SNX5. A single ~55 kDa component, the expected size of SNX5, was detected at abundant levels in untreated primary macrophages (Fig. 1B). Macropinocytosis is known to be down-regulated in macrophages by the treatment with 10 ng/ml lipopolysaccharide (LPS) [31]. We examined the number of macropinosomes formed during a 3 min pulse labelling with dextran using an automated image analysis protocol described in the methods. Untreated BMMs had 723 macropinosomes per 100 cells (n = 213) compared to 305 macropinosome per 100 cells (n = 182) in BMMs treated with LPS for 24 h (Fig. 1C–D). This 58% reduction in the number of macropinosomes formed is consistent with that observed previously using bulk endocytosis markers [31]. Phalloidin staining of the macrophages indicated that the cells remained healthy after the 24 h LPS treatment. Immunoblotting showed a 40% reduction in total level of SNX5 protein in macrophages after a 24 h LPS treatment (Fig. 1B). This reduced SNX5 level is consistent with previously published microarray data [33]. Endogenous SNX5 is clearly associated with macropinosomes in these BMMs and collectively, this data shows that SNX5 levels correlates with macropinocytic activity.

### SNX5 modulates macropinocytosis

To directly explore the role of SNX5 in macropinocytosis we have used HEK-FlpIn cells which stably express SNX5-GFP. This cell line was the previously described HEK-FlpIn cell line transfected with pcDNA5/FRT-EGFPSNX5, referred to as HEK-GFP-SNX5 [24]. HEK-GFP-SNX5 is a polyclonal cell line where the construct is integrated in a defined chromosomal location and therefore avoids differences due to clonal variation. HEK-GFP-SNX5 has overall ~2 fold-increase in the level of SNX5 compared with HEK-FlpIn cells [25], an increase in SNX5 level which was consistent throughout the course of these studies, as monitored by immunoblotting.

**Figure 2:**
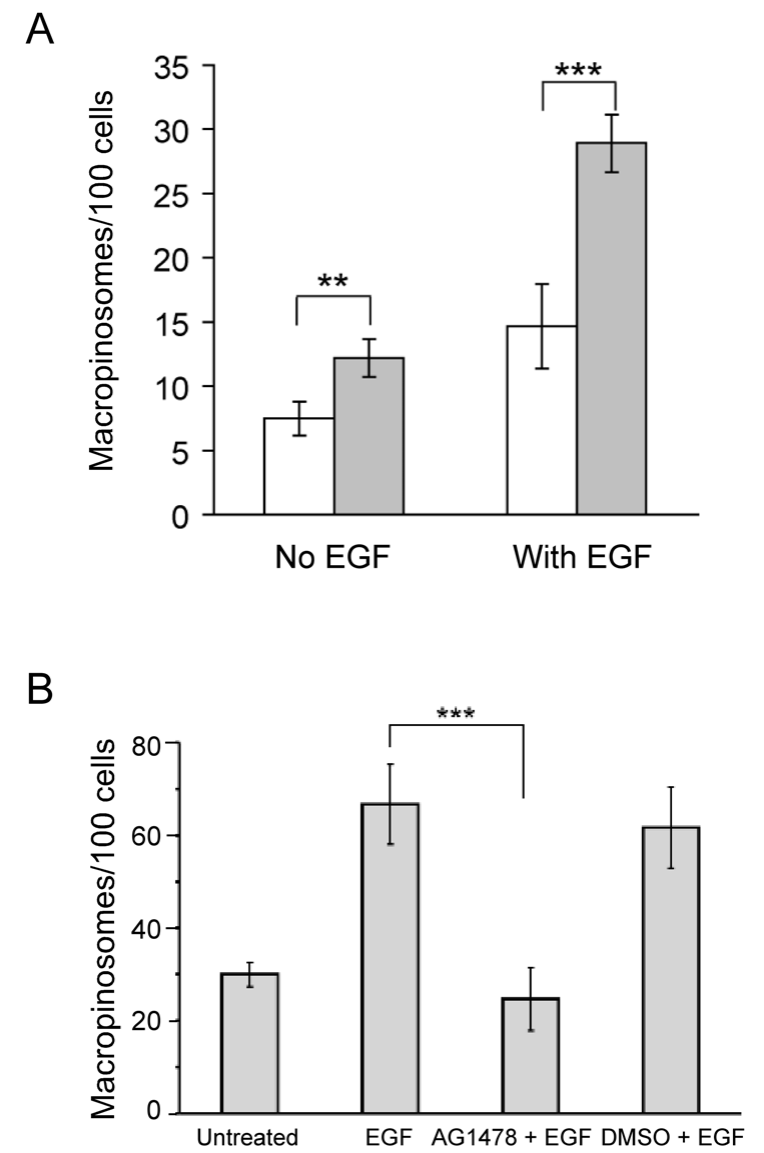
**SNX5 levels influence macropinocytosis activity**. (A) HEK-FlpIn parental (white bars) and HEK-GFP-SNX5 (grey bars) cells were serum-starved for 16 h and pulsed with 100 μg/ml dextran (10,000 Da) conjugated to tetramethylrhodamine for 5 min in the presence or absence of 100 ng/ml EGF at 37°C prior to fixation in 4% PFA. Macropinosomes were identified as dextran-positive structures > 0.5 μm in diameter, and counted using an automated image analysis protocol as described in Methods. The mean number of macropinosomes per 100 cells was determined over 500 cell triplicates for each condition (B) HEK-GFP-SNX5 cells were serum-starved overnight and either left untreated or treated with 100 ng/ml EGF in the presence and absence of 100 nM of AG1478, or in the presence of 0.001% DMSO (carrier control), as indicated. Cells were fixed and permeabilised and stained with anti-human EEA1 and Alexa568-conjugated goat anti-mouse IgG. Macropinosomes were identified as large >1 μm EEA1 positive-structures, as described in text. The mean number of macropinosomes from a triplicate of 100 cells was determined without knowledge of the identity of sample. Each experiment was repeated twice. Shown is mean and error bars represent standard deviation. ** p < 0.05, *** p < 0.005.

Next we investigated if the elevated level of SNX5 in HEK-GFP-SNX5 cells modulated the level of macropinocytosis. To estimate changes in macropinocytosis, newly-formed macropinosomes were labelled using a 5 min pulse of the fluid phase marker dextran (10,000 Da). Only dextran-positive-structures >0.5 μm in diameter were scored as macropinosomes, and this size-based exclusion will minimise any contribution from changes in pinocytosis kinetics. Untreated serum-starved HEK-GFP-SNX5 cells showed a 1.6 fold increase (p < 0.02) in constitutive macropinocytosis compared with parental HEK-FlpIn cells (Fig 2A). Previously we demonstrated that SNX5-positive macropinosomes were co-labelled with EEA1 and dextran [25], and EEA1-positive structures (>1 μm in diameter) were counted as an additional measure of macropinosome number. This assay also identified a 2.3 fold increase in the constitutive levels of macropinocytosis in the HEK-GFP-SNX5 cells (not shown). Together these data show that elevated levels of SNX5 influences the generation of macropinosomes.

We then determined the impact of the elevated level of SNX5 on macropinocytosis after EGF stimulation. After a 5 minute EGF stimulation of serum-starved cells, HEK-GFP-SNX5 cells exhibited a 2.0 fold increase in the number of macropinosomes compared to HEK-FlpIn cells (p < 0.005), utilizing the Dextran-uptake assay (Fig 2A). These data demonstrate that cells expressing elevated levels of SNX5 have an increase in the capacity for macropinocytosis. Moreover, the increase in both constitutive and receptor stimulated-macropinocytosis as a consequence of increased levels of SNX5 indicates that this molecule plays a role in the generation of these endocytic structures.

Next we investigated the relationship between EGF-R signalling and macropinocytosis in the stable HEK-GFP-SNX5 cells using AG1478, an EGF-R specific tyrosine kinase inhibitor that prevents EGF-R activation [34]. Significantly, treatment of cells with AG1478 inhibited the increase in the number of macropinosomes normally observed when cells are treated with EGF alone (Fig. 2B). DMSO, the carrier for AG1478, had no impact on the level of macropinosomes at the same concentration (Fig 2B). Similar results were obtained in three separate experiments. The data demonstrate that the increased macropinocytic activity of HEK-GFP-SNX5 cells following EGF binding is in response to cell signalling events.

**Figure 3:**
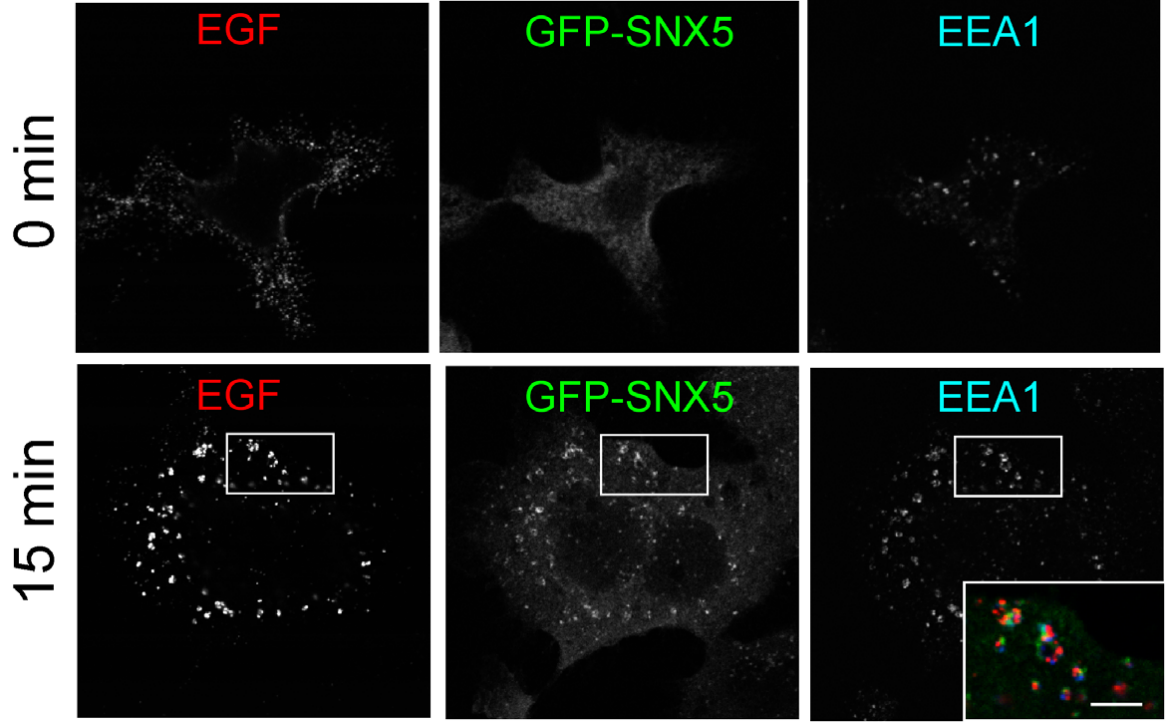
**EGF-R is internalized into macropinosomes in HEK-GFP-SNX5 cells**. (A) HEK-GFP-SNX5 cells were transfected with EGF-R and 24 h later incubated with Alexa555-conjugated EGF on ice. After washing, cells were incubated at 37°C for 15 min, then fixed and permeabilised and stained with human anti-EEA1 antibody followed by Alexa647-conjugated anti-human IgG. Insert shows overlay of GFP, SNX5 and EEA1. In inset, bar = 5 μm.

To determine if the elevated macropinocytic activity induced by increased levels of SNX5 in HEK-GFP-SNX5 cells can support the internalization of EGF-R, we transfected HEK-GFP-SNX5 cells with EGF-R to increase the numbers of receptors for detection by immunofluorescence. Transfected HEK-GFP-SNX5 cells were incubated with Alexa555-conjugated EGF on ice, then the cells washed and incubated at 37°C to allow internalization of surface bound ligand-receptor complexes. As previously reported [25], SNX5 and EEA1 are located to distinct domains of the macropinosome (Fig. 3). After either 5 min (not shown) or 15 min incubation at 37°C, the majority (84%) of SNX5- and EEA1-labeled macropinosomes were also labelled with Alexa555-EGF (Fig. 3), indicating that the activated receptor can be internalized into these large structures and furthermore suggests that the formation of these structures from the plasma membranes occurs in the proximity of activated receptors.

### SNX5 functions in the formation of macropinosomes

**Figure 4:**
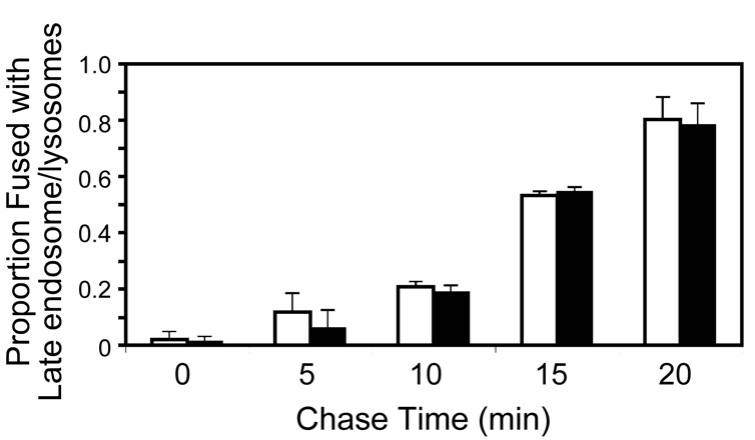
**Elevated SNX5 does not impact on macropinosome maturation**. HEK-FlpIn parental (white bars) or HEK-GFP-SNX5 (black bars) cell monolayers were cultured in the presence of 100 μg/ml Alexa647-conjugated dextran (10,000 MW) for 4 h at 37°C, washed and incubated overnight to label the late endosomes and lysosomes. The cells were then incubated with 100 μg/ml TR-labeled dextran (10,000 MW) for 5 min at 37°C, washed and then incubated at 37°C in serum free media for up to 20 min, as indicated, to internalize the TR-labeled dextran. Monolayers were fixed in 4% PFA and the samples mounted and images captured using a confocal microscope. The proportion of macropinosomes that had fused and acquired content from the late endosomes/lysosomes was scored.

Upon EGF-R activation, SNX5 is recruited to the plasma membrane and the newly-formed macropinosomes [24] and is clearly associated during the early maturation stages of individual macropinosomes [25]. Therefore, SNX5 may function to drive the formation of new macropinosomes and/or to regulate the maturation of the macropinosomes. To determine if elevated levels of SNX5 in the HEK-GFP-SNX5 cells influence the rate of maturation of macropinosomes, we performed experiments to determine if the delivery of macropinosome contents to the late endosome/lysosome was altered. The content mixing assay described by Luzio and colleagues was used [29] where cells were first exposed to Alexa647-conjugated dextran as a fluid phase marker to label late endosomes and lysosomes and then the Alexa647-positive cells incubated in TR-labelled dextran for 5 min, washed and analyzed over the subsequent 20 min for mixing of the two fluorochromes as an indication of fusion of the newly-formed macropinosomes with late endosome/lysosome. No difference was detected in the proportion of macropinosomes that acquired Alexa647-dextran between parental and HEK-GFP-SNX5 cells (Fig. 4), indicating that the increased levels of SNX5 does not affect macropinosome maturation rates. This result indicates that the observed increase in macropinosome number in HEK-GFP-SNX5 cells is not due to a delay in the kinetics of maturation. To further compare the maturation kinetics between the two cells lines we examined the rates of EGF-R degradation. Immunoblotting of the endogenous EGF-R was performed to determine the level of receptor at various time-points after EGF treatment. Importantly the level of the EGF-R observed prior to the addition of EGF was the same between both cell lines (not shown). No significant difference was observed in the rate at which EGF-R was degraded in the HEK-GFP-SNX5 cells when compared to the parental cell line (not shown). These two observations combined indicate that the kinetics of macropinosome maturation was not altered by the increased level of SNX5.

**Figure 5:**
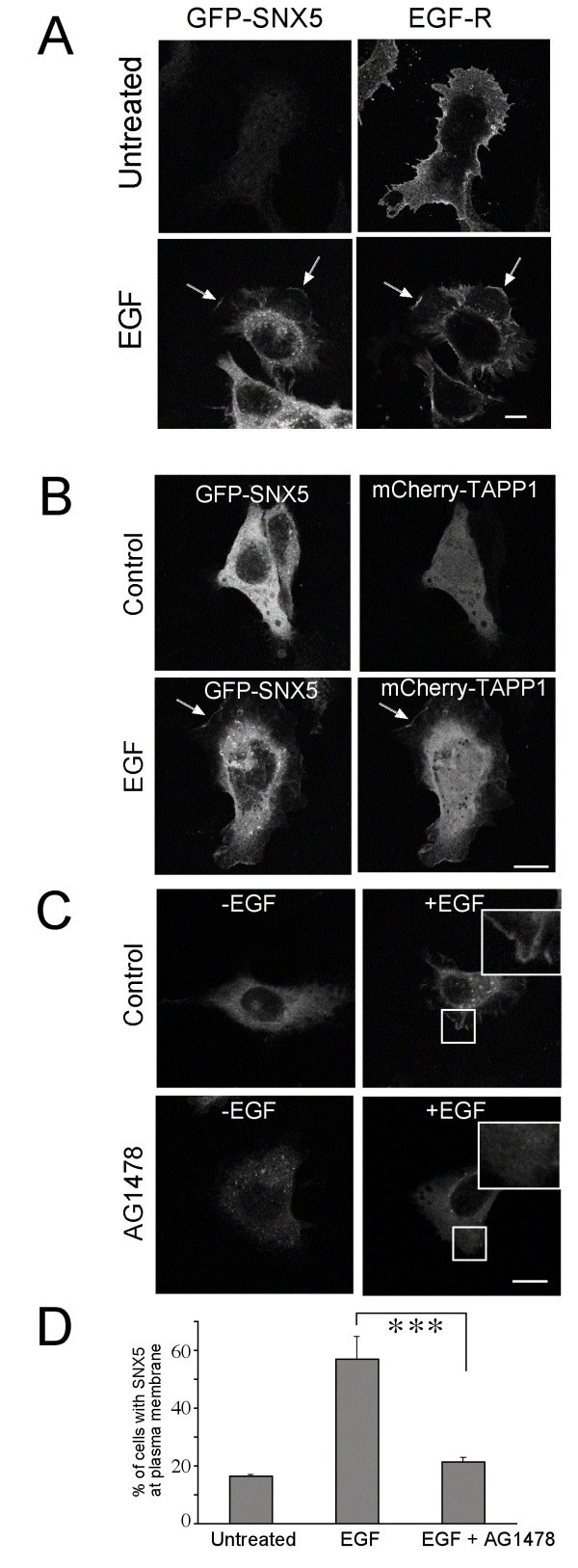
**SNX5 is recruited to regions of the plasma membrane rich in PI(3,4)P_2_**. (A) HEK-GFP-SNX5 cells were transfected with EGF-R and 24 h later serum-starved overnight and either left untreated or treated with 100 ng/ml EGF for 3 min at 37°C, as indicated. Cells were fixed and stained with anti-human EGF-R antibody followed by Alexa568-conjugated goat anti-mouse IgG for detection of surface EGF-R. Optical sections were taken to highlight the plasma membrane. Arrows indicate membrane ruffles showing co-localization of GFP-SNX5 and EGF-R (B) HEK-GFP-SNX5 cells were transfected with mCherry-TAPP1-PH and 24 h later serum-starved overnight then either left untreated or treated with 100 ng/ml EGF for 5 min at 37°C and then fixed. Arrows indicate co-localization of GFP-SNX5 and mCherry-TAPP1-PH (C) HEK-GFP-SNX5 cells were serum-starved overnight and either left untreated or treated with 100 ng/ml EGF for 3 min at 37°C, in the presence or absence of 100 nM AG1478, as indicated, and fixed. Boxed images are shown magnified on upper right hand corner. (D). Cells from (C) were scored for plasma membrane GFP-SNX5 by epifluorescence microscopy. 100 cells in triplicate were analysed. Shown is mean and error bars represent standard deviation. *** p <0.005. Bars = 10 μm.

Next we investigated if SNX5 recruited to the plasma membrane after EGF stimulation can influence the rate of formation of macropinosomes. To determine the spatial relationship between plasma membrane recruitment and the location of EGF-R, serum-starved HEK-GFP-SNX5 cells were treated with EGF for 3 min and non-permeabilised cells stained with anti-EGF-R antibodies. Although the staining intensity of the endogenous cell surface EGF-R was weak, the receptor was detected in the ruffling edges of the cell and showed a co-localization with GFP-SNX5 (not shown). To enhance the sensitivity, this experiment was repeated with transfected HEK-GFP-SNX5 cells expressing elevated levels of EGF-R (Fig. 5A). GFP-SNX5 was not detected on the plasma membrane in serum-starved EGF-R transfected cells prior to EGF treatment, however, following EGF treatment cell surface GFP-SNX5 was clearly concentrated in the ruffling edges of the cell and showed a striking co-localization with surface EGF-R (Fig. 5A).

Activation of EGF-R results in the production of PtdIns(3,4,5)*P*_3 _and its breakdown product, PtdIns(3,4)*P*_2_. We have shown that the PX domain of SNX5 binds to both PtdIns(3)*P *as well as PtdIns(3,4)*P*_2_. To determine the relationship of PtdIns(3,4)*P*_2 _generated from activated EGF-R and the plasma membrane recruitment of SNX5, we have used the PtdIns(3,4)*P*_2_-specific probe, mCherry-TAPP1-PH domain [35], to determine the location of PtdIns(3,4)*P*_2 _at the plasma membrane. HEK-GFP-SNX5 cells were transfected with mCherry-TAPP1-PH domain, then the cells serum-starved and treated with EGF for 5 min. mCherry-TAPP1-PH was located on the plasma membrane of EGF-stimulated cells, but not non-stimulated cells, and showed co-localization with GFP-SNX5, demonstrating that SNX5 was recruited to regions of the plasma membrane rich in PtdIns(3,4)*P*_2 _after EGF stimulation (Fig. 5B). Significantly, treatment of EGF-stimulated cells with AG1478 resulted in the inhibition of SNX5 recruitment to the plasma membrane (Fig. 5C). Qualitative cell analysis showed a reduction from ~57% to 20% of cells with detectable SNX5 at the cell surface (Fig. 5C). The residual 20% positive cells represents a level similar to that observed in non-stimulated cells. Therefore, inhibition of EGF-R tyrosine kinase activity by AG1478 inhibits the recruitment of SNX5 to the plasma membrane and the generation of macropinosomes. Taken together we propose that activation of EGF-R and its effector kinase PI3K are required for SNX5 recruitment to the plasma membrane and subsequent generation of macropinosomes that internalize receptor-EGF complexes.

## Discussion

The mechanisms that drive macropinocytosis are poorly defined. Our previous studies showed that SNX5 associates with newly-formed macropinosomes following EGF stimulation [25]. Given the potential of sorting nexins to have membrane binding and bending properties, mediated by the PX and BAR domains, SNX5 represented a potential regulator of this endocytic process. SNX5 is recruited to the plasma membrane following receptor stimulation and is associated with newly-formed macropinosomes derived from membrane ruffles. In this study we have analysed the impact of SNX5 on numerous aspects of the macropinocytic process. Our results demonstrate that SNX5 is present in abundant levels in primary macrophages that possess high intrinsic macropinocytic activity and that on reduction in macropinocytosis, cell levels of SNX5 are reduced also. Moreover, stable overexpression in HEK293 cells resulted in the recruitment of SNX5 to the cell surface following EGF stimulation and in a significant increase in the number of newly-formed macropinosomes. Upon abrogation of the cell-surface recruitment of SNX5 via inhibition of EGF-R tyrosine kinase activity, the generation of macropinosomes is substantially reduced. Collectively the data indicates SNX5 is a molecular component of macropinocytosis specifically involved in the biogenesis and formation of macropinosomes.

The PX domain of SNX5 has the capacity to bind both PtdIns(3)*P *and PtdIns(3,4)*P*_2. _[24]. The PI binding specificity of SNX5 contrasts with SNX1 which binds selectively PtdIns(3)*P *and PtdIns(3,5)*P*_2, _also based on liposome binding assays [36]. The plasma membrane recruitment of SNX5 following EGF stimulation is likely to be dependent on an interaction with PtdIns(3,4)*P*_2_. Cell signalling is essential for the recruitment of SNX5 to the cytosolic face of the plasma membrane, as AG1478 inhibited the movement of SNX5 to the plasma membrane, presumably due to the lack of PtdIns(3,4,5)*P*_3 _and its breakdown product PtdIns(3,4)*P*_2._. However, AG1478 does not prevent EGF-R internalization via the clathrin-dependent pathway (unpublished observations), indicating, at least in this system that receptor dimerization is sufficient for clathrin-mediated endocytosis, and consistent with previous findings [37].

Given the association of EGF signalling and the behaviour of SNX5, it is formally possible that SNX5 is modulating the EGF-R signalling cascade and thereby influencing macropinocytosis indirectly. However, our data argues for a direct role of SNX5 in enhancing the early events of macropinocytosis rather than potentiating EGF-R signalling. Firstly, constitutive macropinocytosis in the absence of EGF stimulation is increased >2 fold in HEK-GFP-SNX5 cells compared with parental HEK-FlpIn cells. Secondly, the kinetics of macropinosome maturation and the rate of EGF-R degradation is similar in parental and HEK-GFP-SNX5 cells following EGF stimulation, indicating SNX5 is promoting the early events driving the biogenesis of macropinosomes, but does not influence the rate of maturation and delivery to lysosomes.

A number of the SNXs can form homodimers and heterodimers *in vitro *and *in vivo*. As SNX5 has a coiled-coil C-terminal and a predicted BAR domain, it is likely that it exists as a dimer. We have previously demonstrated that SNX5 does not form a homodimer but can associate with SNX1 and that association of SNX5 with endosomes appears to be dependent on the formation of SNX5/SNX1 heterodimers [25]. SNX5 is selectively recruited to membrane ruffles of activated cells and is not associated with SNX1 [24]. Therefore it is likely that SNX5 exists in two pools, one pool independent of SNX1 and another pool as a heterodimer with SNX1. We propose that a SNX1-independent pool of SNX5 is recruited to the plasma membrane to initiate the formation of macropinosomes whereas the SNX5/SNX1 pool is recruited to a subdomain of maturing macropinosomes to promote tubulation. Furthermore, the differential recruitment of SNX5 to either plasma membrane or nascent macropinosomes is likely to be dependent on the specificity of phosphoinositide binding. Clearly further studies are required to identify the partners of SNX5.

## Conclusion

Based on our published data [24,25] and the data above, our model for macropinosome maturation is as follows. Following receptor stimulation, GFP-SNX5 is recruited to discrete patches on the cytoplasmic surface of the plasma membrane via its phosphoinositide binding PX domain. There is a direct relationship between the level of cell surface SNX5 protein and macropinocytic activity, highlighting the role for this sorting nexin in driving this signal-activated endocytosis pathway. Newly-formed SNX5-positive macropinosomes recruit EEA1 and the retromer complex, possibly mediated by PtdIns(3)*P *arising from the hydrolysis of PtdIns(3,4)*P*_2_. The BAR domain of SNX5, in a complex with other sorting nexins, has the potential to distort the limiting membrane of the macropinosome into extensive tubular elements that subsequently separate from the macropinosome and traffic in a microtubule-dependent manner to perinuclear endosomes. As the tubular structures depart from the macropinosome there is a reduction in the surface area of the macropinosome, which matures into a Rab7-positive structure and eventually fuses with late endosomes/lysosomes. The identification of machinery that regulates this process now provides the ability to directly assess the biological role of macropinocytosis in a range of physiological settings.

## Abbreviations

M-CSF: macrophage colony-stimulating factor; EGF: epidermal growth factor; SNX5: sorting nexin 5; TR-dextran: dextran conjugated to tetramethylrhodamine; GFP: green fluorescent protein; BMMs: bone marrow-derived macrophages; LPS: lipopolysaccharide; HEK-GFP-SNX5: HEK293 cells stably expressing SNX5-GFP; PtdIns: phosphoinositides; PX: phox domain; PFA: paraformaldehyde

## Authors' contributions

JPL generated the anti-mouse SNX5 antibodies and carried out the macropinosome analysis and inhibition assays. JTHW performed the dextran uptake and the computational quantitation of macropinosome formation. MCK carried out the macrophage analyses and macropinosome maturation assays. RDT participated in the design and co-ordination of the study and helped to draft the paper. PAG participated in the design and co-ordination of the study and drafted the paper. All authors read and approved the final manuscript.

## References

[B1] Hewlett LJ, Prescott AR, Watts C (1994). The coated pit and macropinocytic pathways serve distinct endosome populations. J Cell Biol.

[B2] Swanson JA, Watts C (1995). Macropinocytosis. Trends Cell Biol.

[B3] Haigler HT, McKanna JA, Cohen S (1979). Rapid stimulation of pinocytosis in human carcinoma cells A-431 by epidermal growth factor. J Cell Biol.

[B4] Racoosin EL, Swanson JA (1992). M-CSF-induced macropinocytosis increases solute endocytosis but not receptor-mediated endocytosis in mouse macrophages. J Cell Sci.

[B5] Sun P, Yamamoto H, Suetsugu S, Miki H, Takenawa T, Endo T (2003). Small GTPase Rah/Rab34 is associated with membrane ruffles and macropinosomes and promotes macropinosome formation. J Biol Chem.

[B6] West MA, Bretscher MS, Watts C (1989). Distinct endocytotic pathways in epidermal growth factor-stimulated human carcinoma A431 cells. J Cell Biol.

[B7] Owen DJ, Evans PR (1998). A structural explanation for the recognition of tyrosine-based endocytotic signals. Science.

[B8] Sallusto F, Lanzavecchia A (1995). Dendritic cells use macropinocytosis and the mannose receptor to concentrate antigen to the MHC class II compartment. J Exp Med.

[B9] Steinman RM, Brodie SE, Cohn ZA (1976). Membrane flow during pinocytosis. A stereologic analysis. J Cell Biol.

[B10] Norbury CC (2006). Drinking a lot is good for dendritic cells. Immunology.

[B11] West MA, Wallin RPA, Matthews SP, Svensson HG, Zaru R, Ljunggren H-G, Prescott AR, Watt C (2004). Enhanced dendritic cell antigen capture via toll-like receptor induced actin remodelling. Science.

[B12] Carpentier JL, Lew DP, Paccaud JP, Gil R, Iacopetta B, Kazatchkine M, Stendahl O, Pozzan T (1991). Internalization pathway of C3b receptors in human neutrophils and its transmodulation by chemoattractant receptors stimulation. Cell regulation.

[B13] Alpuche-Aranda CM, Racoosin EL, Swanson JA, Miller SI (1994). Salmonella stimulate macrophage macropinocytosis and persist within spacious phagosomes. J Exp Med.

[B14] Hooshmand-Rad R, Claesson-Welsh L, Wennstrom S, Yokote K, Siegbahn A, Heldin CH (1997). Involvement of phosphatidylinositide 3'-kinase and Rac in platelet-derived growth factor-induced actin reorganization and chemotaxis. Exp Cell Res.

[B15] Pitt A, Mayorga LS, Stahl PD, Schwartz AL (1992). Alterations in the protein composition of maturing phagosomes. J Clin Invest.

[B16] Kimber WA, Deak M, Prescott AR, Alessi DR (2003). Interaction of the protein tyrosine phosphatase PTPL1 with the PtdIns(3,4)P2-binding adaptor protein TAPP1. Biochem J.

[B17] Cantley LC (2002). The phosphoinositide 3-kinase pathway. Science.

[B18] Cronin TC, DiNitto JP, Czech MP, Lambright DG (2004). Structural determinants of phosphoinositide selectivity in splice variants of Grp1 family PH domains. Embo J.

[B19] Worby CA, Dixon JE (2002). Sorting out the cellular functions of sorting nexins. Nat Rev Mol Cell Biol.

[B20] Carlton J, Bujny M, Rutherford A, Cullen P (2005). Sorting nexins – unifying trends and new perspectives. Traffic.

[B21] Haft CR, de la Luz Sierra M, Barr VA, Haft DH, Taylor SI (1998). Identification of a family of sorting nexin molecules and characterization of their association with receptors. Mol Cell Biol.

[B22] Habermann B (2004). The BAR-domain family of proteins: a case of bending and binding?. EMBO Rep.

[B23] Teasdale RD, Loci D, Houghton F, Karlsson L, Gleeson PA (2001). A large family of endosome-localized proteins related to sorting nexin 1. Biochem J.

[B24] Merino-Trigo A, Kerr MC, Houghton F, Lindberg A, Mitchell C, Teasdale RD, Gleeson PA (2004). Sorting nexin 5 is localized to a subdomain of the early endosomes and is recruited to the plasma membrane following EGF stimulation. J Cell Sci.

[B25] Kerr MC, Lindsay MR, Luetterforst R, Hamilton N, Simpson F, Parton RG, Gleeson PA, Teasdale RD (2006). Visualisation of macropinosome maturation by the recruitment of sorting nexins. J Cell Sci.

[B26] Kawamoto T, Sato JD, Le A, Polikoff J, Sato GH, Mendelsohn J (1983). Growth stimulation of A431 cells by epidermal growth factor: identification of high-affinity receptors for epidermal growth factor by an anti-receptor monoclonal antibody. Proc Natl Acad Sci USA.

[B27] Sester DP, Brion K, Trieu A, Goodridge HS, Roberts TL, Dunn J, Hume DA, Stacey KJ, Sweet MJ (2006). CpG DNA activates survival in murine macrophages through TLR9 and the phosphatidylinositol 3-kinase-Akt pathway. J Immunol.

[B28] Kjer-Nielsen L, van Vliet C, Erlich R, Toh BH, Gleeson PA (1999). The Golgi-targeting sequence of the peripheral membrane protein p230. J Cell Sci.

[B29] Bright NA, Gratian MJ, Luzio JP (2005). Endocytic delivery to lysosomes mediated by concurrent fusion and kissing events in living cells. Curr Biol.

[B30] Gleeson PA, Anderson TJ, Stow JL, Griffiths G, Toh BH, Matheson F (1996). p230 is associated with vesicles budding from the trans-Golgi network. J Cell Sci.

[B31] Tsang AW, Oestergaard K, Myers JT, Swanson JA (2000). Altered membrane trafficking in activated bone marrow-derived macrophages. Journal of leukocyte biology.

[B32] Su AI, Wiltshire T, Batalov S, Lapp H, Ching KA, Block D, Zhang J, Soden R, Hayakawa M, Kreiman G (2004). A gene atlas of the mouse and human protein-encoding transcriptomes. Proc Natl Acad Sci USA.

[B33] Hume DA, Wells CA, Ravasi T (2007). Transcriptional regulatory networks in macrophages. Novartis Foundation symposium.

[B34] Levitzki A, Gazit A (1995). Tyrosine kinase inhibition: an approach to drug development. Science.

[B35] Ivetac I, Munday AD, Kisseleva MV, Zhang XM, Luff S, Tiganis T, Whisstock JC, Rowe T, Majerus PW, Mitchell CA (2005). The type Ialpha inositol polyphosphate 4-phosphatase generates and terminates phosphoinositide 3-kinase signals on endosomes and the plasma membrane. Mol Biol Cell.

[B36] Cozier GE, Carlton J, McGregor AH, Gleeson PA, Teasdale RD, Mellor H, Cullen PJ (2002). The phox homology (PX) domain-dependent, 3-phosphoinositide-mediated association of sorting nexin-1 with an early sorting endosomal compartment is required for its ability to regulate epidermal growth factor receptor degradation. J Biol Chem.

[B37] Wang Q, Villeneuve G, Wang Z (2005). Control of epidermal growth factor receptor endocytosis by receptor dimerization, rather than receptor kinase activation. EMBO Rep.

